# A decision tree to identify the combinations of non-communicable diseases that constitute the highest risk for dental caries experience: A hospital records-based study

**DOI:** 10.1371/journal.pone.0257079

**Published:** 2021-10-06

**Authors:** Hyun-Joo Kim, Youngseuk Cho, Yunhwan Noh, Ji-Young Joo, Hae Ryoun Park

**Affiliations:** 1 Department of Periodontology, Dental and Life Science Institute, Pusan National University, School of Dentistry, Yangsan, Republic of Korea; 2 Department of Periodontology and Dental Research Institute, Pusan National University Dental Hospital, Yangsan, Republic of Korea; 3 Periodontal Disease Signaling Network Research Center, School of Dentistry, Pusan National University, Yangsan, Republic of Korea; 4 Department of Statistics, Pusan National University, Busan, Republic of Korea; 5 Department of Oral Pathology, School of Dentistry, Pusan National University, Yangsan, Republic of Korea; University Lyon 1 Faculty of Dental Medicine, FRANCE

## Abstract

To investigate whether dental status, represented by the DMFT score, was affected by the presence of NCDs and determined the NCDs that had a greater impact on the DMFT score. This retrospective cross-sectional study included a total of 10,017 individuals. The presence of NCDs was investigated based on self-reported medical history recorded on each patient’s dental hospital record. Individual DMFT score was evaluated on the basis of the dental records and panoramic radiographs. The data were further analyzed using multiple regression analysis and chi-squared automatic interaction detection (CHAID) analysis. A total of 5,388 individuals had more than one NCD among hypertension (HT), diabetes mellitus (DM), hyperlipidemia, cardiovascular disease (CVD), and osteoporosis. The average DMFT score was 8.62 ± 7.10 in the NCD group, significantly higher than that in those without NCD (5.53 ± 5.48) (*P* < 0.001). In the regression analysis, age, NCDs, and psychiatric problems were selected as risk factors of DMFT score. In the CHAID decision tree analysis, age was the risk factor that most influenced the DMFT score. HT was the most influential factor in a newly generated decision tree excluding age, and osteoporosis, DM, and CVD were important risk factors acting in the subgroups. Patients with NCD had worse dental conditions than those who did not, and some combinations of NCDs related highest risk for a dental caries-related index. In clinical practice, dentists should provide meticulous care for dental caries in elderly patients with NCDs, especially when certain diseases, such as HT, osteoporosis, DM, and CVD, are present together.

## Introduction

Non-communicable diseases (NCDs) are not transmitted directly from one person to another and usually present as chronic inflammatory disorders, which progress slowly over a long period of time. NCDs include cardiovascular disease (CVD), cancer, diabetes, chronic respiratory disease, Alzheimer’s disease, and osteoporosis. They are the leading cause of morbidity, accounting for 72% of all deaths worldwide, and this proportion is growing [1]. Several international organizations, medical associations, and global philanthropies have implemented various global action plans for prevention and control of NCDs, especially in low-income and middle-income countries [2]. The core strategies of these policies usually focus on blocking behavioral and metabolic problems that are known to be major risk factors for NCDs, such as smoking, unhealthy diets, physical inactivity, obesity, and hazardous alcohol intake [3].

Oral diseases are a global public health problem with major health and economic implications, affecting over 3.5 billion people worldwide. The most prevalent oral diseases are dental caries and periodontal disease [4]. The initiation and progression of these diseases in each individual is influenced by multiple and diverse combinations of several factors, including inherited factors such as genetic variants and acquired factors such as social, economic, educational, local environment, and lifestyle-related factors [5]. If left untreated, these diseases can lead to tooth loss, which can reduce masticatory function, cause nutritional problems, and pose a threat to general health. Oral conditions disproportionately affect the poor and socially disadvantaged members of societies, particularly in low-income and middle-income countries.

NCDs and oral diseases have several characteristics in common, since they are all multifactorial, chronic, and progressive. In particular, oral diseases share some major risk factors with NCDs associated with excessive sugar consumption, such as diabetes and obesity [6]. Moreover, oral diseases are chronic inflammatory conditions in nature, and therefore impose a long-lasting inflammatory burden on the whole body. For these reasons, research on the bidirectional relationship between oral diseases and other NCDs has received increased attention in recent years [7–9]. While most studies have demonstrated plausible relationships between them, the actual causes and mechanisms underlying these relationships remain to be elucidated.

Most of the related studies to date have focused on elucidating the relationship between NCDs and periodontitis among oral diseases [10–12]. Thus, the correlations between NCDs and other tooth-related diseases, such as caries, retained roots, and missing teeth, have received relatively less attention. The most commonly used caries index is the DMFT (decayed, missing, filled teeth) score, which counts the number of decayed, missing, and filled teeth due to dental caries [13]. This index is based on an individual’s past and present caries experience, and reflects the dental health status. Data mining is useful to extract useful information from large databases and visualize it so that it can be easily interpreted, and the decision tree has recently gained prominence as the most effective method for data mining in medical research [14, 15]. Therefore, this study aimed to build a prediction model using Chi-square automatic interaction detection (CHAID) analysis (a decision tree algorithm) of a large-scale sample to identify the NCDs that had a greater impact on the DMFT score.

## Material and methods

The protocol of this study was approved by the Institutional Review Board of Pusan National University Dental Hospital (PNUDH-2019-047). In this study, no consent form was obtained as data were analyzed retrospectively and anonymously based on hospital records.

### Study design and data collection

This retrospective cross-sectional study was conducted using data obtained from a hospital (Pusan National University Dental Hospital, Yangsan city, Korea) chart review and examination of dental panoramic radiographs. Among the subjects who visited the Department of Periodontics between 2014 and 2019, a total of 10,017 individuals (5,255 female and 4,792 male) who underwent panoramic radiography on the first day of their hospital visit were included in this study. All procedures outlined below were investigated and recorded by one experienced dentist.

The presence of NCDs was investigated on the basis of self-reported medical history (PMH) recorded on the initial chart of each patient’s electronic dentistry record (EDR). Generally, only the medical histories of the patient diagnosed by the relevant specialist were recorded in the PMH of the initial examination record. The following diseases were considered as NCDs: hypertension (HT), diabetes mellitus (DM), hyperlipidemia, cardiovascular disease (CVD), osteoporosis, cancer, thyroid disease, liver disease, arthritis, respiratory disease, renal disease, and dementia. Clear infectious diseases were not considered NCDs, and patients with more than one disease were included in the classification of each disease in duplicate or more, depending on the number of diseases.

The patient’s dental status was examined based on the EDR and panoramic radiographs. The numbers of total, decayed (DT), missing (MT), filled (FT), and teeth with C4 caries (teeth showing destruction of the entire tooth crown) were assessed. The number of DTs included teeth with advanced caries to the extent that they could be clearly distinguished on radiographs. Teeth with filling or restorative material that could be seen on radiographs were counted as FTs. Teeth filled with temporary restorations recorded in EDR were classified as DTs. The DMFT score was calculated by summing up the numbers of DTs, MTs, and FTs. DMFT rates were calculated as a percentage by dividing the DMFT score by the number of teeth.

### Sample size determination

The sample size was established considering an effect size of 0.002, a significant level of 0.05, and a power of 95% in regression analysis (G*power ver. 3.1.9.7). It was determined that approximately 9,896 subjects would be needed. A total of 10,017 subjects enrolled to this study.

### Statistical analysis

Data are presented as mean ± standard deviation for continuous variables and as numbers and percentages for categorical variables. Descriptive statistics were generated for general health variables and variables related to dental status. An independent t-test was used to analyze the significant differences between the variables representing dental status in the group according to the presence of NCDs. A multiple regression analysis was performed to explore the significance of age, sex, NCDs, smoking, and psychiatric problem, as predictor variables for the DMFT scores. Statistical significance was set at *p* < 0.05.

CHAID analysis, a decision tree algorithm, was used to identify the most important risk factors associated with NCDs from a pool of several potential risk factors that were extracted by reviewing EDRs. The decision tree was created using the following procedure: the most significant risk factor (the one with the largest Chi-square value) divides the entire patient population into ≥2 subgroups. These groups are subsequently subdivided by the next most significant risk factor. The analysis continues in this step-by-step manner to select the most influential variable at each stage until there are no more significant risk factors [16]. In other words, one node was separated if the *p* value met the adjusted significance value (*p* < 0.05), and conversely, if not, it was considered a terminal node. Analyses were performed using SPSS software (IBM Corp. 23.0, Armonk, NY, USA).

## Results

### Baseline characteristics of the study population

Data based on hospital records from 10,017 patients were included in this analysis. The baseline characteristics of the patients are presented in Table 1. The mean age of the patients was 52.91 ± 12.69 years. Of the 10,017 patients, 4,792 were male, and 5,225 were female, with a male:female ratio of 0.91:1. Among the patients included, 5,388 (53.8%) had more than one NCD. The most common NCDs were HT (2,139, 21.3%), DM (1,067, 10.6%), hyperlipidemia (882, 8.8%), CVD (799, 8.0%), osteoporosis (397, 4.0%), and other diseases, including cancer (387, 3.8%), liver disease (336, 3,4%), arthritis (220, 2.2%), respiratory disease (147, 1,4%), and dementia (45, 0.4%). Among the selected participants, 845 patients were smokers, while 233 had been diagnosed with psychiatric problems such as depression and sleep disorders.

**Table 1:** Patient characteristics.

	Subjects (n = 10017)
Age, mean ± SD	52.91 ± 12.69
Sex	
Male, n (%)	4792 (47.8)
Female, n (%)	5225 (52.2)
NCD, n (%)	5388 (53.8)
hypertension	2139 (21.3)
diabetes mellitus	1067 (10.6)
hyperlipidemia	882 (8.8)
cardiovascular disease	799 (8.0)
osteoporosis	397 (4.0)
cancer	387 (3.9)
thyroid disease	384 (3.8)
liver disease	336 (3.4)
arthritis	220 (2.2)
respiratory disease	147 (1.5)
renal disease	144 (1.4)
dementia	45 (0.4)
Smoking, n (%)	845 (8.4)
Psychiatric problem, n (%)	233 (2.3)

### Dental status according to the presence of NCDs

After grouping patients according to the presence or absence of NCDs, the number of residual teeth, DTs, MTs, FTs, DMFT score, and teeth with C4 caries were investigated and analyzed (Table 2). The average number of residual teeth was 23.83 ± 5.05 and 25.57 ± 3.57 in patients with and without NCDs, respectively. The average DMFT score and DMFT rate were 8.62 ± 7.10 and 30.80 ± 25.38 in patients with NCDs, respectively, which were significantly higher than those in patients without NCDs (5.53 ± 5.48 and 19.74 ± 19.60, respectively). In patients with NCDs, the number of teeth that progressed to C4 caries was 0.15 ± 0.76, which was also significantly higher than that in patients without NCDs (0.07 ± 0.45). In other words, patients with NCDs had fewer residual teeth, higher values for all DMFT-related indices, and more permanent teeth with severe caries experience, such as root rest, than patients without NCDs, and these data indicated that their teeth health status was worse than that of patients without NCDs.

**Table 2:** Analysis of dental status according to the presence of NCDs.

	NCD		P value
	Yes (n = 5388)	No (n = 4629)	
Residual teeth (n, mean ± SD)	23.83 ± 5.05	25.57 ± 3.57	< 0.001
DT (n, mean ± SD)	0.76 ± 1.73	0.48 ± 1.16	< 0.001
MT (n, mean ± SD)	4.17 ± 5.05	2.43 ± 3.57	< 0.001
FT (n, mean ± SD)	3.69 ± 3.22	2.62 ± 2.80	< 0.001
DMFT (n, mean ± SD)	8.62 ± 7.10	5.53 ± 5.48	< 0.001
DMFT rate (%, mean ± SD)	30.80 ± 25.38	19.74 ± 19.60	< 0.001
C4 caries (n, mean ± SD)	0.15 ± 0.76	0.07 ± 0.45	< 0.001

DT: decayed teeth; MT: missing teeth; FT: filled teeth; DMFT: the sum of DT, MT, and FT; DMFT rate: (DMFT score/ the number of teeth) x 100.

### Multiple regression analysis to identify the relationship between risk factors and DMFT scores

Multiple regression analysis was performed to analyze the relationship between DMFT and sex, age, smoking, psychiatric problems, and presence of NCDs, which are information that can be obtained from EDR (Table 3). As a result of analysis of all subjects, it was found that among the risk factors, age, presence of NCDs, and psychiatric problem significantly affected DMFT scores. In other words, it was found that the DMFT score increased with increasing age, with NCDs, or with psychiatric problems. According to the WHO quantification for DMFT (1986), DMFT 4.5 or higher is classified as high risk. Therefore, we separated subjects with a DMFT score of 4.5 or higher and re-evaluated the relationship between risk factors and DMFT score. The risk factors found to have a significant relationship with DMFT scores were the same as the results of the previous analysis for all subjects, and the relevance was also the same.

**Table 3:** Multiple regression analysis of effect of risk factors on DMFT score.

Dependent variable	Predictors	Unstandardized Coefficients		Standardized coefficients	t	Sig.
		B	Sta. Error	beta		
DMFT score	Sex (M)	-0.035	0.119	-0.003	-0.297	0.766
	Age	0.231	0.005	0.453	45.958	< 0.001
	Smoking	0.158	0.216	0.007	0.734	0.463
R^2^ = 0.222	Psychiatric problem	1.575	0.385	0.037	4.095	< 0.001
	NCDs	0.446	0.129	0.034	3.446	0.001
	constant	-5.477	0.314		-17.421	< 0.001
DMFT score (≥ 4.5)	Sex (M)	0.090	0.151	0.008	0.601	0.548
	Age	0.197	0.007	0.383	27.993	< 0.001
	Smoking	0.258	0.287	0.012	0.899	0.368
R^2^ = 0.160	Psychiatric problem	1.614	0.449	0.045	3.496	< 0.001
	NCDs	0.327	0.161	0.028	2.028	0.043
	constant	-0.368	0.448		-0.822	0.411

### Decision tree analysis to identify the predictors related to DMFT scores

#### Decision tree analysis of all subjects

Using the CHAID algorithm, we generated a decision tree to determine the priority of factors related to the DMFT score. The purpose of the analysis was to identify the most important factors among the potential risk factors based on the collected data, such as age, sex, type of NCD (including HT, DM, hyperlipidemia, CVD, osteoporosis, cancer, thyroid disease, liver disease, arthritis, respiratory disease, renal disease, and dementia), smoking, and psychiatric problems. The maximum number of nodes and maximum depth were not limited, and the minimum size of each node was also not set.

**Fig 1:**
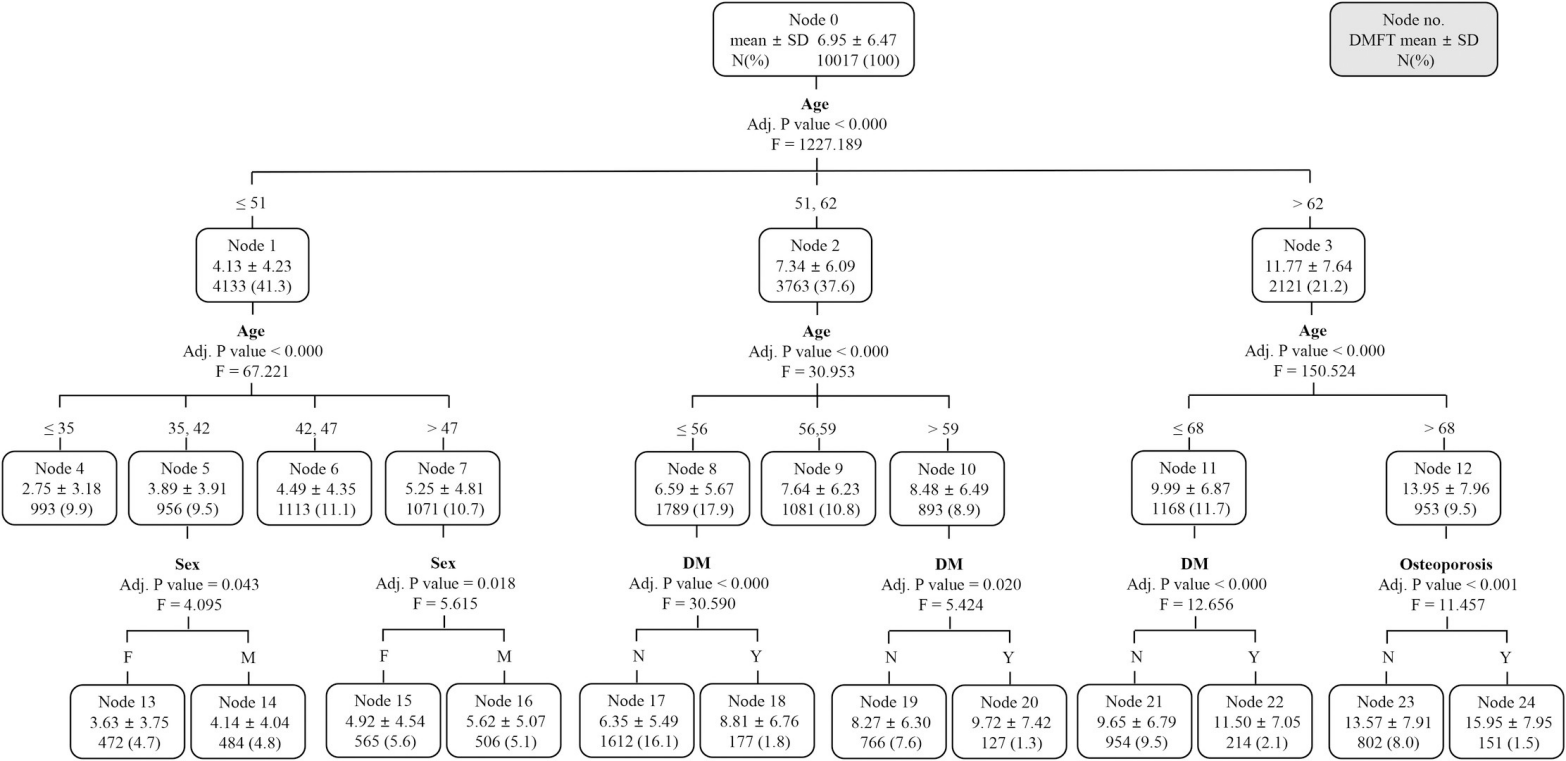
A chi-square automatic interaction detection classification tree analysis to identify the predictors related to the DMFT of all subjects. Data were presented as mean ± standard deviation. F, female; M, male; DM, diabetes mellitus.

All individuals were divided into 14 subgroups through different branches from the root node to the leaf node (Fig 1). The mean DMFT score varied from 2.75 to 15.95. Among the potential risk factors, the top-level node of the CHAID classification decision tree (primary split) was “age.” It divided the patients into three subgroups based on the age interval of 51 to 62 years, and the DMFT scores were 4.13 ± 4.23, 7.34 ± 6.09, and 11.77 ± 7.64 for each age subgroup (age ≤ 51 years, 51 years < age ≤ 62 years, 62 years < age). The next decision split was also based on age. The subgroups were divided by different factors at the third-level split: sex, DM, and osteoporosis. In patients aged below 51 years, sex was the factor of the third split in some of the subgroups, and the DMFT score was higher in men in the same age group. For the age groups of 51–56, 59–62, and 62–68 years, the third-level split was based on DM status, and the DMFT score was higher in patients with DM. For patients aged > 68 years, osteoporosis was the third-level split factor. In particular, if the patient was over 68 years old and had osteoporosis, the DMFT score was 15.95 ± 7.95, the highest among the 14 subgroups.

**Fig 2:**
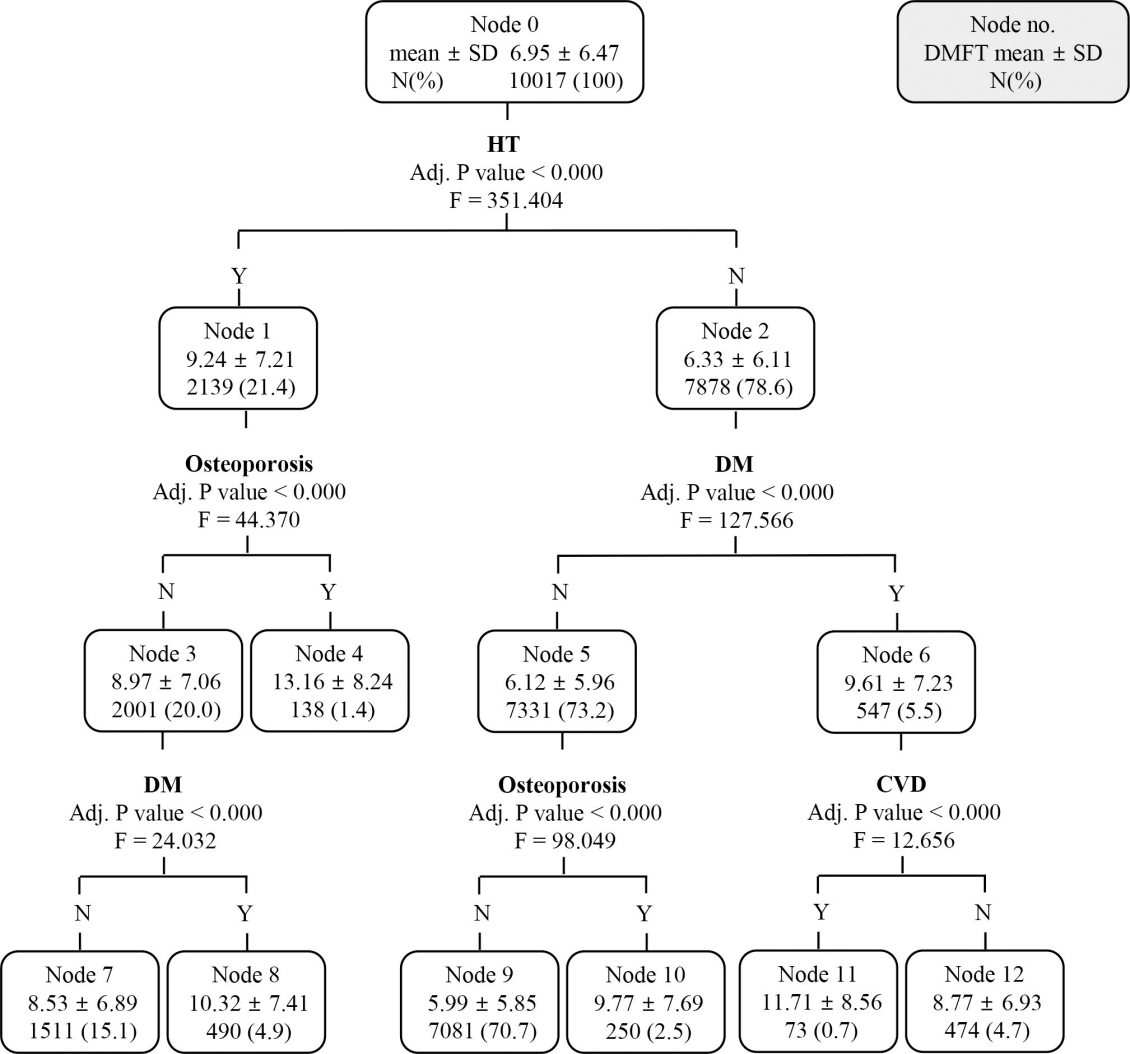
A chi-square automatic interaction detection classification tree analysis to identify the predictors related to the DMFT of all subjects; excluding age. Data were presented as mean ± standard deviation. HT, hypertension; DM, diabetes mellitus; CVD, cardiovascular disease.

Considering the finding that the top two levels in Fig 1 were split by age, we generated a new decision tree model using CHAID by excluding age among the candidate risk factors. In this tree, all patients were split by seven terminal nodes through different branches from the root node to the leaf node, with the average DMFT score varying from 5.99 ± 5.85 to 13.16 ± 8.24 (Fig 2). In the newly created decision tree, the first decision split was based on HT, and the patients with HT had a DMFT score of 9.24 ± 7.21, which was about three more compared to that of patients without HT. The patients in node 1 (with HT) were split according to osteoporosis at the second-level split, and the DMFT score was higher in osteoporosis patients. At the third-level split, patients with HT and osteoporosis were no longer split, but patients with HT without osteoporosis were re-split according to DM status. The individual in node 8 (with HT, without osteoporosis, and with DM) had a DMFT score of 10.32 ± 7.41, which was higher than that of node 7 (with HT, without osteoporosis, and without DM). HT-free patients on the right side of the decision tree were split by DM status at the second level. In the subsequent third-level split, DM-free patients were separated by osteoporosis, and those with DM were separated by CVD status. In the Fig 2 decision tree, in all nodes, the presence of a disease corresponded to a higher average DMFT score. Among the 7 terminal nodes, the average DMFT score (13.16 ± 8.24) was the highest among patients with HT and osteoporosis (node 4), which was about twice the average for all patients (node 0, 6.95 ± 6.47).

#### Decision tree analysis of subjects with high risk of caries (DMFT score ≥ 4.5)

**Fig 3:**
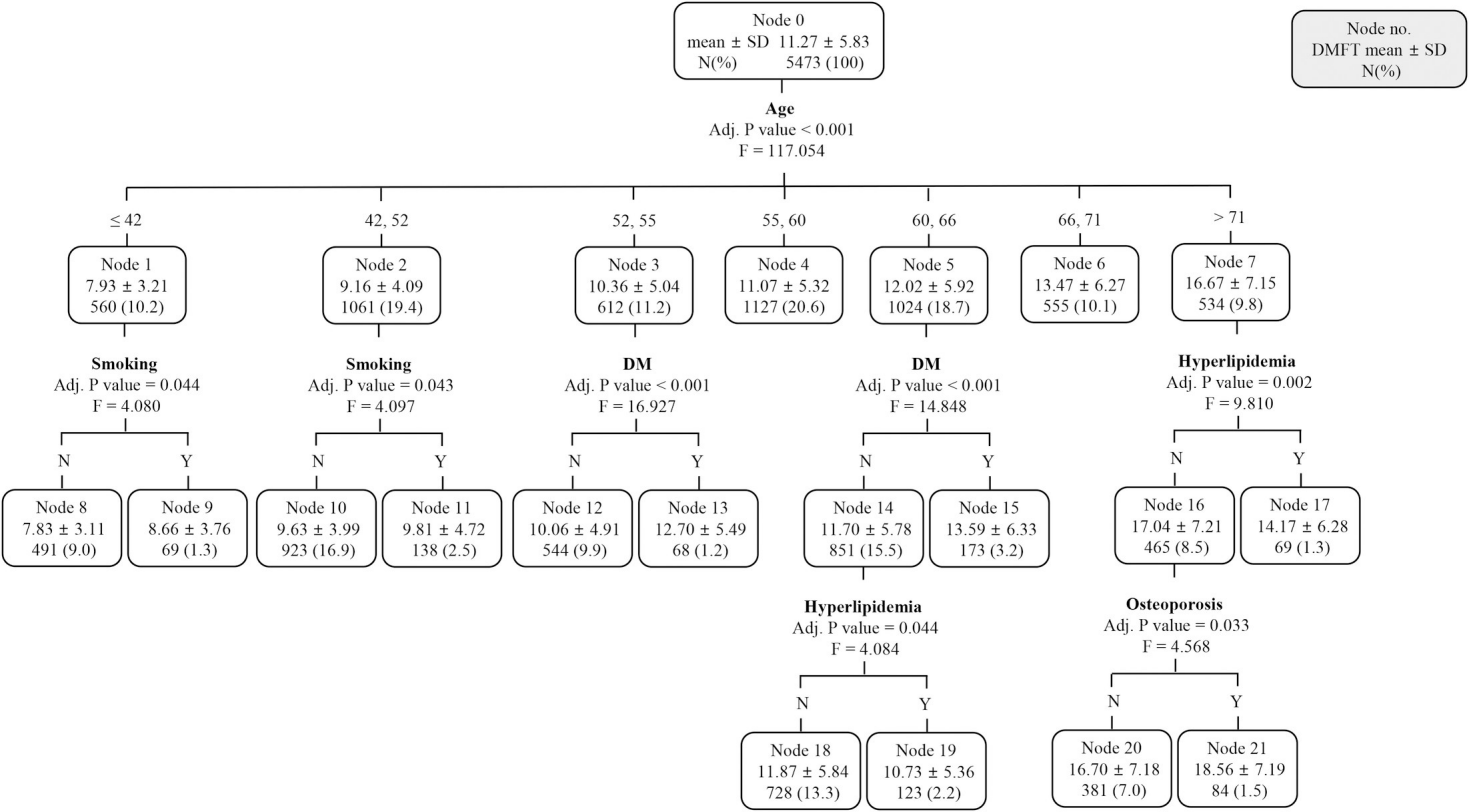
A chi-square automatic interaction detection classification tree analysis to identify the predictors related to the DMFT of subjects with DMFT score ≥ 4.5. Data were presented as mean ± standard deviation. F, female; M, male; DM, diabetes mellitus.

Decision tree analysis was performed on subjects (n: 5,473) with a high risk of caries with a DMFT score ≥ 4.5, and their DMFT score was 11.27 ± 5.83 (Fig 3). Like the decision tree for all subjects (Fig 1), the first split factor was age, which divided node 0 into 7 subgroups. The second split factor was smoking in those under 52 years of age, DM in those aged 52–55 and 60–66, and hyperlipidemia in those over 71 years of age. Subjects aged 60–66 years without DM were re-separated by the third split factor, hyperlipidemia, and DMFT was higher in the absence of hyperlipidemia. Subjects aged > 71 years and without hyperlipidemia, it was separated by the third level split factor, osteoporosis, and in these subjects (over 71 years of age, without hyperlipidemia, and with osteoporosis), the DMFT score was the highest with 18.56 ± 7.19.

**Fig 4:**
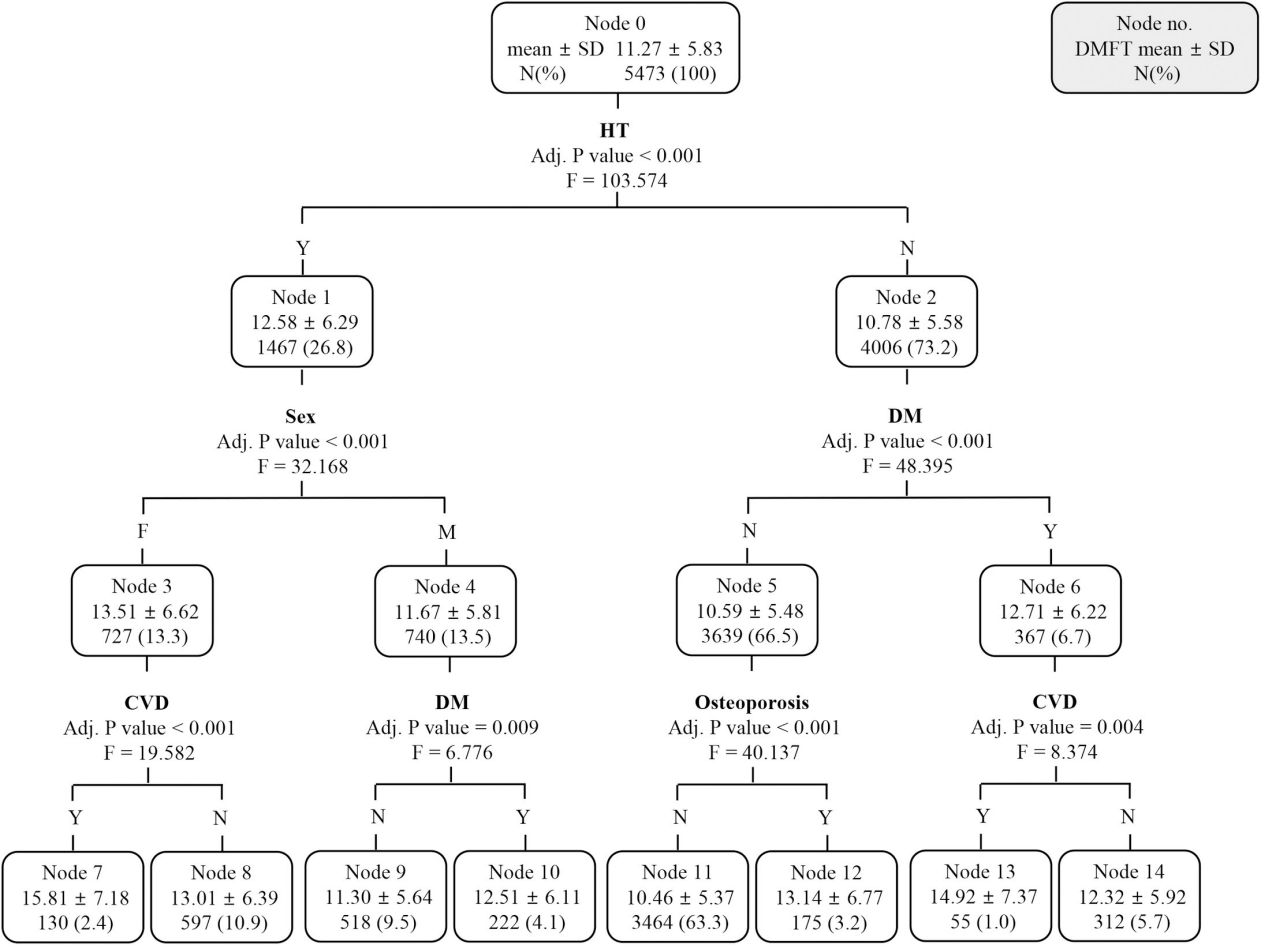
A chi-square automatic interaction detection classification tree analysis to identify the predictors related to the DMFT of subjects with DMFT score ≥ 4.5; excluding age. Data were presented as mean ± standard deviation. HT, hypertension; F, female; M, male; DM, diabetes mellitus; CVD, cardiovascular disease.

In subjects at high risk of caries, another decision tree model was newly generated by excluding age in the same way as the analysis for all subjects (Fig 4). They were divided by HT at the first level, and the second split factor was sex in the presence of HT and DM in the absence of HT. At the third level, node 3 (female subjects with HT) was re-separated by CVD, node 4 (male subjects with HT) by DM, node 5 (subjects without HT and DM) by osteoporosis, and node 6 (subject without HT and with DM) by CVD, respectively. Female subjects with HT and CVD (node 7) had the highest DMFT scores (15.81 ± 7.18).

## Discussion

One of the most common oral diseases, dental caries, is characterized as a biofilm-related, sugar-driven, multifactorial, and dynamic disease. Modifications in lifestyle, diet, and behavioral factors are known to affect not only the occurrence of new lesions, but also the potential for progression of existing lesions [5]. Therefore, proper control of the multiple factors affecting dental caries is important to effectively reduce its prevalence. In contrast to the extensive literature on periodontitis, only some studies have attempted to determine the correlations between dental status and various systemic diseases [17, 18], but a clear causal relationship has not yet been established since a wide range of genetic predispositions to behavioral traits are involved as common risk factors for these diseases [7]. To our knowledge, no previous study has assessed the effects of multiple NCDs, not each disease, on dental conditions in more than 10,000 patients. In addition to the type of NCD, age, sex, smoking, and psychiatric problems were also included as risk factor candidates in the present study, and the priority of these factors in terms of their impact on the DMFT score was assessed using a decision tree algorithm.

The results of our study suggest that in patients with at least one NCD, all of the indices reflecting the dental status showed a significantly negative trend, i.e., the number of residual teeth was lower and the number of caries-experienced teeth was higher. These findings confirmed a correlation between the dental status and NCDs. In one study on the possible link between dental diseases and atherosclerosis in patients on hemodialysis, C4 teeth, the MT index, and the DMFT score were significantly higher in the patient group. On the other hand, there was no difference in periodontal pocket depth regardless of systemic disease [19]. In particular, with regard to tooth loss, a recent meta-analysis that included more than 8 million patients reported that the loss of one tooth increases the risk of both coronary heart disease and stroke by 1.5% each [20]. This was also demonstrated in another study that indicated a significant relationship between oral health and CVD by demonstrating that patients with more than 18 missing teeth had a 2.5-fold higher risk of CVD [18]. The long-term drug intake required for systemic disease control in patients with NCDs can cause reduced saliva flow and dry mouth, leading to oral microbial dysbiosis, which can be detrimental to oral health [21]. Oral microbial dysbiosis has been recently recognized as an essential factor in the initiation of dental caries and periodontitis, which are two representative oral diseases [22, 23].

The prevalence of NCDs and oral diseases is usually known to increase with age because of their chronic and progressive nature. In our data analysis using a decision tree algorithm to determine the priority of DMFT-related factors, age was the most influential factor among the risk factor candidates. According to one study that examined the DMFT score in Korean elderly patients, the oral health status, including the DMFT score and the numbers of missing and residual teeth, significantly differed between elderly and aged individuals. Therefore, age could be a strong risk factor for tooth loss among elderly people [24]. Physiological age-driven changes, such as a decrease in salivation, changes in salivary composition, and immunosenescence, eventually disrupt the homeostasis of the oral microbiome [25]. In addition, environmental age-driven changes such as gingival recession and the presence of complicated dental restorations make oral hygiene management more difficult. A combination of these factors can lead to an increase in the DMFT score with age.

HT, osteoporosis, DM, and CVD were decisive factors when the decision tree was newly generated (Fig 2) in all subjects by excluding age from the candidate risk factors to rule out the effect of age on the DMFT score. Of course, these are the high-ranked diseases in patients with more than one NCDs, but the fact that they did not include hyperlipidemia, the third most common disease in patients with NCDs, suggests that these relationships are not simply based on disease frequency. Among the seven terminal nodes in the newly created decision tree, the DMFT score was the highest among HT patients with osteoporosis, which was about twice as much as that of the fewest cases. In addition, the group that showed the highest DMFT score among the 14 terminal nodes in the first generated decision tree (Fig 1), which included age, consisted of patients over 68 years of age with osteoporosis. This was similarly observed in the decision tree (Figs 3 and 4) regenerated by separating subjects with high caries risk (DMFT score > 4.5). Vascular tissue and bone appear to share some common mechanisms in the regulation of the skeletal and cardiovascular systems [26], and the pathophysiological link between HT and osteoporosis has been supported by several biological and epidemiological studies [27, 28]. Moreover, deterioration of diet quality can act as a common link connecting dental caries, HT, and osteoporosis. In particular, the concentrations of calcium, phosphate, and vitamin D are known to be involved in the regulation of bone metabolism and blood pressure, and are reported to affect the DMFT score depending on the concentrations in the dental plaque [29, 30]. This study confirmed that the simultaneous presence of HT and osteoporosis acts as a strong risk factor affecting the DMFT score through the various connectable routes discussed above.

Of the seven terminal nodes of the newly created decision tree (Fig 2) in all subjects, the group with the second-highest DMFT was the group with both DM and CVD among patients without HT. This was also founded in the decision tree (Fig 4) regenerated by excluding age in the high caries risk subjects. The strong relationship between DM and CVD has been a topic of concern among clinicians and researchers, and it involves a complex interplay between numerous pathophysiologic mechanisms, including abnormalities of glucose homeostasis, which can trigger several alterations in cardiovascular structure and function [31]. Moreover, the CVD risk in diabetic patients is significantly modified by a variety of genetic factors, such as variations in haptoglobin genotypes and apolipoprotein E gene, and epigenetic factors [32, 33]. Among these factors, glucose homeostasis has also been reported to correlate with the DMFT score [34]. A recent study reported that tooth loss could be a good predictor of CVD risk [35], and our study demonstrated that the simultaneous presence of CVDs and DMs contributed to a high DMFT score, confirming the presence of inverse relationships as well.

CHAID analysis was performed to prioritize factors affecting the DMFT score in subjects with high caries risk (DMFT score > 4.5). As with all subjects analyzed previously, age was the first split factor, but did not act as the second level split factor. It can be inferred that the higher the DMFT score, that is, the higher the caries risk, the relatively reduced the effect of age on the DMFT score. Age, DM, and osteoporosis acted as split factors in all subjects and subjects with high caries risk, whereas smoking and hyperlipidemia acted as split factors only in subjects with high caries risk. Smoking acted as a second-level split factor under the age of 52, which is a relatively younger age group compared to other groups, and it can be inferred that the effect of smoking was relatively emphasized due to the low frequency of NCDs in this group. Smoking was not classified as a risk factor in regression analysis, but acted as a split factor in decision tree analysis. This suggests that smoking alone is not a high risk factor for DMFT, but may act as a risk factor when combined with other factors. When decision tree regenerated except for age in subjects at high risk for caries, HT, CVD, DM, and osteoporosis acted as major spilt factors as in all subjects. In this decision tree (Fig 4) the patients with HT and CVD showed the highest DMFT score, similarly to the patients with HT and osteoporosis in decision tree for all subjects (Fig 2). The subgroup with the second highest DMFT score was the group with both DM and CVD among patients without HT in both decision tree (Figs 2 and 4). Through this, it was found that HT, CVD, DM, and osteoporosis act as major risk factors for the DMFT score regardless of the high risk of caries.

## Conclusion

In conclusion, in this large hospital record-based cohort study using decision tree algorithms, we found that NCD patients had worse dental status than healthy subjects, and age and combinations of some NCDs, such as HT, osteoporosis, DM, and CVD, could act as strong risk factors for dental caries indicators. In clinical practice, dentists need to be more active in the treatment of patients with NCDs, especially those simultaneously presenting with HT, osteoporosis, DM, and CVD at the same time, to prevent tooth loss due to dental caries.

## Supporting information

S1 Data (XLSX)

## References

[1] Global, regional, and national life expectancy, all-cause mortality, and cause-specific mortality for 249 causes of death, 1980–2015: a systematic analysis for the Global Burden of Disease Study 2015. Lancet (London, England). 2016;388:1459–1544. 10.1016/S0140-6736(16)31012-1 PMC5388903 27733281

[2] NCD Countdown 2030: worldwide trends in non-communicable disease mortality and progress towards Sustainable Development Goal target 3.4. Lancet (London, England). 2018;392:1072–1088. doi: 10.1016/S0140-6736(18)31992-5 30264707

[3] Beaglehole R, Bonita R, Horton R, Adams C, Alleyne G, Asaria P, et al. Priority actions for the non-communicable disease crisis. Lancet (London, England). 2011;377:1438–1447. doi: 10.1016/S0140-6736(11)60393-0 21474174

[4] Watt RG, Daly B, Allison P, Macpherson LMD, Venturelli R, Listl S, et al. Ending the neglect of global oral health: time for radical action. Lancet (London, England). 2019;394:261–272. doi: 10.1016/S0140-6736(19)31133-X 31327370

[5] Chapple IL, Bouchard P, Cagetti MG, Campus G, Carra MC, Cocco F, et al. Interaction of lifestyle, behaviour or systemic diseases with dental caries and periodontal diseases: consensus report of group 2 of the joint EFP/ORCA workshop on the boundaries between caries and periodontal diseases. J Clin Periodontol. 2017;44 Suppl 18:S39–S51. doi: 10.1111/jcpe.12685 28266114

[6] Peres MA, Macpherson LMD, Weyant RJ, Daly B, Venturelli R, Mathur MR, et al. Oral diseases: a global public health challenge. Lancet (London, England). 2019;394:249–260. doi: 10.1016/S0140-6736(19)31146-8 31327369

[7] Dörfer C, Benz C, Aida J, Campard G. The relationship of oral health with general health and NCDs: a brief review. Int Dent J. 2017;67 Suppl 2:14–18. doi: 10.1111/idj.12360 29023744 PMC9378917

[8] Gondivkar SM, Gadbail AR, Gondivkar RS, Sarode SC, Sarode GS, Patil S, et al. Nutrition and oral health. Dis Mon. 2019;65:147–154. doi: 10.1016/j.disamonth.2018.09.009 30293649

[9] Kedar A, Hariprasad R, Kumar V, Dhanasekaran K, Mehrotra R. Association of metabolic NCD risk factors with oral, breast and cervical precancers and cancers in India. Fam Med Community Health. 2019;7:e000180. doi: 10.1136/fmch-2019-000180 32148727 PMC6910767

[10] Bokhari SA, Khan AA, Butt AK, Hanif M, Izhar M, Tatakis DN, et al. Periodontitis in coronary heart disease patients: strong association between bleeding on probing and systemic biomarkers. J Clin Periodontol 2014;41:1048–1054. doi: 10.1111/jcpe.12284 24946826

[11] Deas DE, Mackey SA, McDonnell HT. Systemic disease and periodontitis: manifestations of neutrophil dysfunction. Periodontol 2000. 2003;32:82–104. doi: 10.1046/j.0906-6713.2003.03207.x 12756035

[12] Hegde R, Awan KH. Effects of periodontal disease on systemic health. Dis Mon. 2019;65:185–192. doi: 10.1016/j.disamonth.2018.09.011 30384973

[13] Broadbent JM, Thomson WM. For debate: problems with the DMF index pertinent to dental caries data analysis. Community Dent Oral Epidemiol. 2005;33:400–409. doi: 10.1111/j.1600-0528.2005.00259.x 16262607 PMC1388190

[14] Atieh MA, Pang JK, Lian K, Wong S, Tawse-Smith A, Ma S, et al. Predicting peri-implant disease: Chi-square automatic interaction detection (CHAID) decision tree analysis of risk indicators. J Periodontol. 2019;90:834–846. doi: 10.1002/JPER.17-0501 30730061

[15] Song YY, Lu Y. Decision tree methods: applications for classification and prediction. Shanghai Arch Psychiatry. 2015;27:130–135. doi: 10.11919/j.issn.1002-0829.215044 26120265 PMC4466856

[16] Che D, Liu Q, Rasheed K, Tao X. Decision tree and ensemble learning algorithms with their applications in bioinformatics. Adv Exp Med Biol. 2011;696:191–199. doi: 10.1007/978-1-4419-7046-6_19 21431559

[17] Dar-Odeh N, Borzangy S, Babkair H, Farghal L, Shahin G, Fadhlalmawla S, et al. Association of Dental Caries, Retained Roots, and Missing Teeth with Physical Status, Diabetes Mellitus and Hypertension in Women of the Reproductive Age. Int J Environ Res Public Health. 2019;16. 10.3390/ijerph16142565 PMC6678296 31323793

[18] De Angelis F, Basili S, Giovanni F, Dan Trifan P, Di Carlo S, Manzon L. Influence of the oral status on cardiovascular diseases in an older Italian population. Int J Immunopathol Pharmacol. 2018;32:394632017751786. doi: 10.1177/0394632017751786 29363361 PMC5849242

[19] Misaki T, Fukunaga A, Shimizu Y, Ishikawa A, Nakano K. Possible link between dental diseases and arteriosclerosis in patients on hemodialysis. PloS one. 2019;14:e0225038. doi: 10.1371/journal.pone.0225038 31834880 PMC6910673

[20] Cheng F, Zhang M, Wang Q, Xu H, Dong X, Gao Z, et al. Tooth loss and risk of cardiovascular disease and stroke: A dose-response meta analysis of prospective cohort studies. PloS one. 2018;13:e0194563. doi: 10.1371/journal.pone.0194563 29590166 PMC5874035

[21] Wolff A, Joshi RK, Ekström J, Aframian D, Pedersen AM, Proctor G, et al. A Guide to Medications Inducing Salivary Gland Dysfunction, Xerostomia, and Subjective Sialorrhea: A Systematic Review Sponsored by the World Workshop on Oral Medicine VI. Drugs R D. 2017;17:1–28. doi: 10.1007/s40268-016-0153-9 27853957 PMC5318321

[22] Hajishengallis G. Immunomicrobial pathogenesis of periodontitis: keystones, pathobionts, and host response. Trends Immunol. 2014;35:3–11. doi: 10.1016/j.it.2013.09.001 24269668 PMC3947349

[23] Rosier BT, Marsh PD, Mira A. Resilience of the Oral Microbiota in Health: Mechanisms That Prevent Dysbiosis. J Dental Res. 2018;97:371–380. doi: 10.1177/0022034517742139 29195050

[24] Chung SY, Song KB, Lee SG, Choi YH. The strength of age effect on tooth loss and periodontal condition in Korean elderly. Arch Gerontol Geriatr. 2011;53:e243–248. doi: 10.1016/j.archger.2011.04.021 21641050

[25] Belibasakis GN. Microbiological changes of the ageing oral cavity. Arch Oral Biol. 2018;96:230–232. doi: 10.1016/j.archoralbio.2018.10.001 30308473

[26] McFarlane SI, Muniyappa R, Shin JJ, Bahtiyar G, Sowers JR. Osteoporosis and cardiovascular disease: brittle bones and boned arteries, is there a link? Endocrine. 2004;23:1–10. doi: 10.1385/ENDO:23:1:01 15034190

[27] Jung MH, Youn HJ, Ihm SH, Jung HO, Hong KS. Heart Rate and Bone Mineral Density in Older Women with Hypertension: Results from the Korea National Health and Nutritional Examination Survey. J Am Geriatr Soc. 2018;66:1144–1150. doi: 10.1111/jgs.15359 29608214

[28] Varenna M, Manara M, Galli L, Binelli L, Zucchi F, Sinigaglia L. The association between osteoporosis and hypertension: the role of a low dairy intake. Calcif Tissue Int. 2013;93:86–92. doi: 10.1007/s00223-013-9731-9 23652773

[29] Antonenko O, Bryk G, Brito G, Pellegrini G, Zeni SN. Oral health in young women having a low calcium and vitamin D nutritional status. Clin Oral Investig. 2015;19:1199–1206. doi: 10.1007/s00784-014-1343-x 25359326

[30] Lin HS, Lin JR, Hu SW, Kuo HC, Yang YH. Association of dietary calcium, phosphorus, and magnesium intake with caries status among schoolchildren. Kaohsiung J Med Sci. 2014;30:206–212. doi: 10.1016/j.kjms.2013.12.002 24656162 PMC11916888

[31] Oktay AA, Akturk HK, Esenboğa K, Javed F, Polin NM, Jahangir E. Pathophysiology and Prevention of Heart Disease in Diabetes Mellitus. Curr Probl Cardiol. 2018;43:68–110. doi: 10.1016/j.cpcardiol.2017.05.001 29471918

[32] Chaudhary R, Likidlilid A, Peerapatdit T, Tresukosol D, Srisuma S, Ratanamaneechat S, et al. Apolipoprotein E gene polymorphism: effects on plasma lipids and risk of type 2 diabetes and coronary artery disease. Cardiovasc Diabetol. 2012;11:36. doi: 10.1186/1475-2840-11-36 22520940 PMC3372424

[33] Costacou T, Ferrell RE, Orchard TJ. Haptoglobin genotype: a determinant of cardiovascular complication risk in type 1 diabetes. Diabetes. 2008;57:1702–1706. doi: 10.2337/db08-0095 18332093

[34] Majbauddin A, Tanimura C, Aoto H, Otani S, Parrenas MCE, Kobayashi N, et al. Association between dental caries indicators and serum glycated hemoglobin-levels among patients with type 2 diabetes mellitus. J Oral Sci. 2019;61:335–342. doi: 10.2334/josnusd.18-0156 31217384

[35] Lee HJ, Choi EK, Park JB, Han KD, Oh S. Tooth Loss Predicts Myocardial Infarction, Heart Failure, Stroke, and Death. J Dent Res. 2019;98:164–170. doi: 10.1177/0022034518814829 30782090

